# Touch, light, wounding: how anaesthetics affect plant sensing abilities

**DOI:** 10.1007/s00299-024-03369-7

**Published:** 2024-11-24

**Authors:** Andrej Pavlovič

**Affiliations:** Department of Biophysics, Faculty of Science, Palacký University, Šlechtitelů 27, 78371 Olomouc, Czech Republic

**Keywords:** Anaesthesia, Carnivorous plant, De-etiolation, Electrical signals, Jasmonates, Wounding

## Abstract

**Key message:**

Anaesthetics affect not only humans and animals but also plants. Plants exposed to certain anaesthetics lose their ability to respond adequately to various stimuli such as touch, injury or light. Available results indicate that anaesthetics modulate ion channel activities in plants, e.g. Ca^2+^ influx.

**Abstract:**

The word anaesthesia means loss of sensation. Plants, as all living creatures, can also sense their environment and they are susceptible to anaesthesia. Although some anaesthetics are often known as drugs with well-defined target to their animal/human receptors, some other are promiscuous in their binding. Both have effects on plants. Application of general volatile anaesthetics (GVAs) inhibits plant responses to different stimuli but also induces strong cellular response. Of particular interest is the ability of GVAs inhibit long-distance electrical and Ca^2+^ signalling probably through inhibition of GLUTAMATE RECEPTOR-LIKE proteins (GLRs), the effect which is surprisingly very similar to inhibition of nerve impulse transmission in animals or human. However, GVAs act also as a stressor for plants and can induce their own Ca^2+^ signature, which strongly reprograms gene expression . Down-regulation of genes encoding enzymes of chlorophyll biosynthesis and pigment-protein complexes are responsible for inhibited de-etiolation and photomorphogenesis. Vesicle trafficking, germination, and circumnutation movement of climbing plants are also strongly inhibited. On the other hand, other cellular processes can be upregulated, for example, heat shock response and generation of reactive oxygen species (ROS). Upregulation of stress response by GVAs results in preconditioning/priming and can be helpful to withstand abiotic stresses in plants. Thus, anaesthetic drugs may become a useful tool for scientists studying plant responses to environmental stimuli.

## Introduction

If you believe it or not, or however fantastic it looks like, plants can be anaesthetized. If you see with your own eyes how anaesthetized carnivorous Venus flytrap plant (*Dionaea muscipula*) is unable to react to the presence of insect prey moving with impunity across the surface of the trap, you realise how strikingly similar this is to the human inability to perceive any stimuli under anaesthesia during surgical operation. But usually, the effect of anaesthesia is much more hidden in plants because they live in a slower time scale and you cannot ask them anything. When anaesthesia research on plants was re-awakened in 2018 (Yokawa et al. 2018), it almost immediately attracted media attention. But the fact that plants can be anaesthetized is old and has already been observed by one of the greatest biologist of all time—Charles Darwin (Darwin 1875). However, the father of plant anaesthesia is considered to be Claude Bernard, who carried out numerous experiments on plants that have often been forgotten (Grémiaux et al. 2014). During recent years, numerous studies about plant anaesthesia have emerged with very interesting results parallels of which can be found in animals and human. Also many comments about plant intelligence and consciousness appeared in response to these discoveries (Chamowitz 2018; Taiz et al. 2019; Draghun et al. 2021; Baluška and Yokawa 2021; Mallatt et al. 2021) indicating that the topic is more provocative than scientific. However, definitely, this is not true. In this review, I would like to avoid any anthropomorphic comparison of plants to animals and humans and describe how anaesthetics affect plant physiology.

## Discovery and definition of anaesthesia

On 16 October 1846, dentist William Thomas Green Morton first publicly demonstrated the use of ether anaesthesia during the painless removal of tumour from the neck of Edward Gilbert Abbott in Massachusetts General Hospital in Boston. The term anaesthesia is derived from the Greek word, which means „*without feeling* “, and was introduced by Oliver Wendell Homes soon after the discovery of etherisation. Diethyl ether dominated general anaesthesia for the first 100 years of its existence and in fact, the term anaesthesia was coined to describe what happens during the process of etherisation (Urban and Bleckwenn 2002; Franks 2006). The discovery of ether anaesthesia was driven by the search for means of eliminating a patient’s pain perception and responses to painful stimuli. Thus, general anaesthetics have been often defined from this anthropocentrism point of view as compounds, which provide immobility, analgesia, amnesia, muscle relaxation, and they induce reversible loss of consciousness at low concentrations (Franks 2006). However such narrow and clinical definitions of the state of anaesthesia are not suitable for other organisms which can be also anaesthetized. In 1878, the French physiologist Claude Bernard published his anaesthesia experiments on plants and concluded that: “*what is alive must sense and can be anaesthetized, the rest is dead*” (Grémiaux et al. 2014). Today, we know that anaesthesia drugs are effective in organisms ranging from paramecia, fungi to plants, to primates and deserve a more generalizable definition (Eckenhoff 2008; Rinaldi 2014; Kelz and Mashour 2019; Adamatzky and Gandia 2022). Kelz and Mashour (2019) defined the state of anaesthesia as disconnection from the environment, both in the receptive (i.e. sensation) and expressive (i.e. response) arms of the interaction. We must also realize that general anaesthetics are not an exclusive human invention for modern medicine, as it is known that plants can produce and emit anaesthetic gases when stressed (i.e. ethylene, divinyl ether, Fammartino et al. 2010; Grémiaux et al. 2014; Yokawa et al. 2019).

### Mechanism of action

Despite the daily use of general anaesthetics in medicine, their mechanism of action is still not clear. How such a diverse group of chemical compounds can induce the same end-points? In 1899 and 1901 Hans Horst Meyer and Charles Ernest Overton published the first experimental evidence of the fact that anaesthetic potency is related to lipid solubility. They assumed that solubilisation of lipophilic general anaesthetic in lipid bilayer of the neuron fluidize membranes, causes its malfunction and anaesthetic effect when critical concentration of anaesthetic is reached (lipid hypothesis of general anaesthesia action, Meyer 1899; Overton 1901). In 1984 Nicholas Franks and Bill Lieb (1984) demonstrated the same correlation for lipid-free protein—firefly luciferase. But because firefly luciferase does not mediate consciousness in human, they suggested that general anaesthetics binds to the hydrophobic pocket of many proteins (protein hypothesis of general anaesthesia, Franks and Lieb 1984). Later, it has been found that also other enzymes are sensitive to anaesthetics, whereas some proteins are sensitive only to certain anaesthetics. For example, the GABA_A_ receptors, which are found throughout the central nervous system, were found to be an anaesthetic target of many general anaesthetics. On the other hand, ketamine is a well-known N-methyl-D-aspartate (NMDA) receptor antagonist (Franks 2006; 2008). However, some general anaesthetics may affect proteins indirectly through the plasma membrane. This modern lipid hypothesis has suggested that anaesthetics disrupt lipid rafts, a region of well-ordered lipids containing glycosphingolipids, cholesterol and protein receptors (Lerner 1997). In the case of TREK-1, activation was shown through an anaesthetic perturbation to membrane lipid clusters and activation of phospholipase D2 (Pavel et al. 2020). In contrast to general anaesthetics, the targets of many local anaesthetics are well-known. Local anaesthetics cause the absence of all sensation (including pain) in a specific body part without loss of consciousness. For example, lidocaine is a well-known inhibitor of sodium channels (Hermanns et al. 2019).

Regardless of which hypothesis for the effect of general anaesthetics is correct, all cellular functions are dependent on proteins and lipid membranes. Nevertheless, the study of the effect of anaesthetics has focused primarily on ion channels. However, no gene mutation of an ion channel has completely abolished the effect of a general anaesthetic, indicating promiscuity of anaesthetic binding. Therefore, potential targets of such pleiotropic molecules are now considered to include, for example, presynaptic neurotransmitter release, the cytoskeleton and mitochondria (in plants, probably also chloroplasts, Nakao et al. 1998, Kelz and Mashour (2019). van Swinderen et al. (1999) has shown that mutations in syntaxin alter sensitivity to general anaesthetics. Syntaxin, together with SNAP-25 and synaptobrevin, form the SNARE complex and regulate presynaptic neurotransmitter release. Hydrophobic general anaesthetics are sequestered in the lipid bilayer and bind directly to the SNARE complex and may interfere with synaptic vesicle fusion (van Snideren and Kottler 2014). Allison and Nunn (1968) have considered microtubules as a possible target of general anaesthesia. Presence of microtubule-stabilizing drugs increased anaesthetic resistance (Emerson et al. 2013). Microtubules and actin control a wide variety processes in the cells. They are important for vesicle transport and fusion, and membrane bioelectricity is also interlinked with the actin cytoskeleton (Baluška and Mancuso 2019). Anaesthetics bind to tubulin, causing microtubules to destabilize (Emerson et al. 2013). They can also cause microtubule-based molecular motors, such as kinesin, to reversibly fall off the microtubule lattice and thus disrupt transport of vesicles, proteins, and organelles (Woll et al. 2018). Mutations of mitochondrial complex I caused increased anaesthetic sensitivity. Because complex I determine the rate of mitochondrial electron transport, inhibition of complex I may have a relatively immediate effect on oxidative phosphorylation within energetically demanding tissues such as the nervous system (Falk et al. 2006). All these targets cross phylogenetic boundaries from nematodes to humans and maybe relevant also for plants (Kelz and Mashour 2019). In the following paragraphs, I will describe how anaesthesia can affect different plant’s responses.

### Touch response

**Fig. 1:**
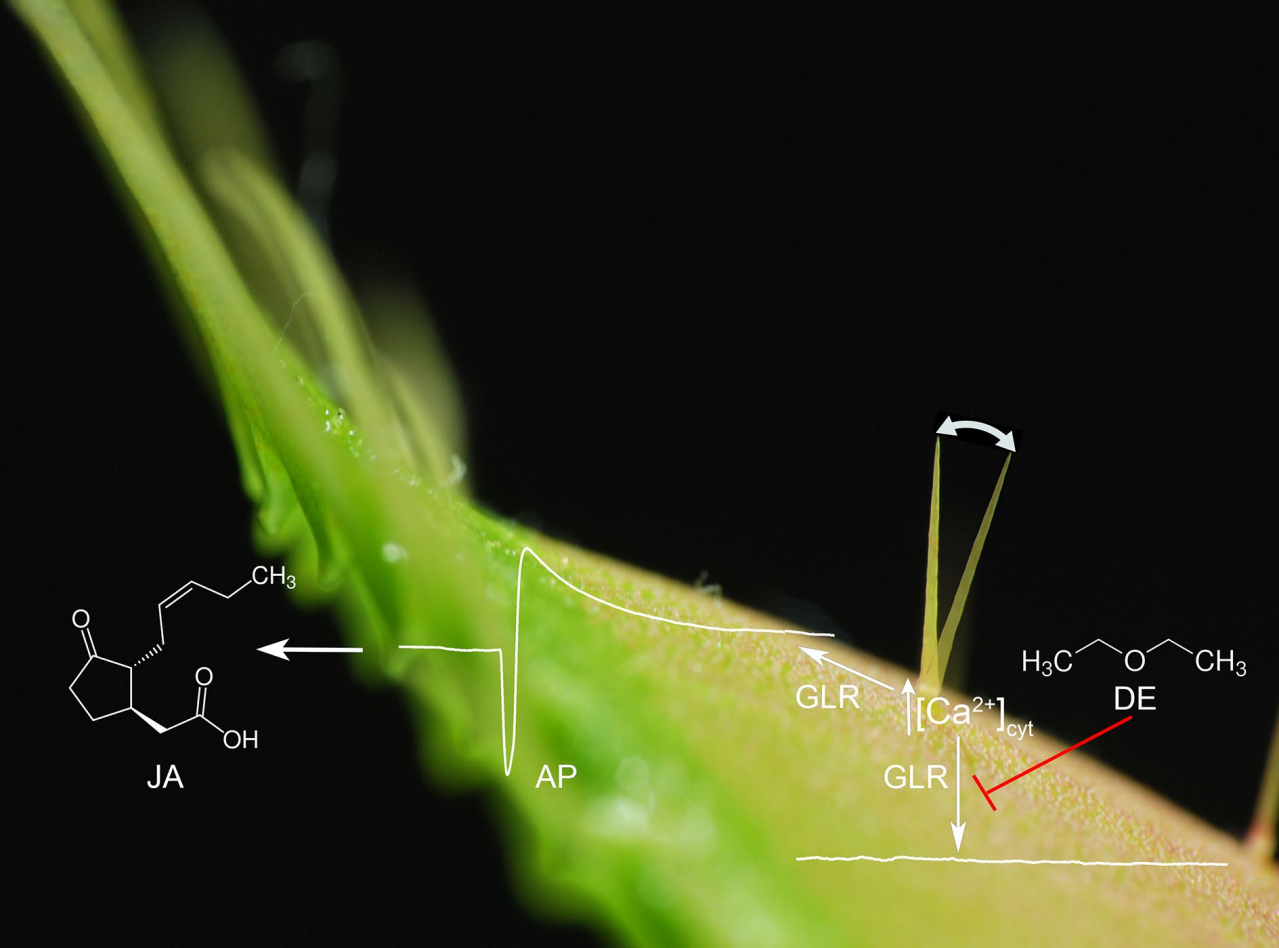
The trap of the carnivorous plant Venus flytrap (*Dionaea muscipula*) cannot snap shut and activate the digestive process under diethyl ether (DE) anaesthesia. Mechanical stimulation of trigger hair in the carnivorous plant Venus flytrap (*Dionaea muscipula*) induces increased intracellular calcium level [Ca^2+^]_cyt_ in podium cells of trigger hair that escapes the trigger hair and propagates along the trap surface in the form of action potential (AP) and Ca^2+^ wave. This propagation is probably GLR-dependent and induces jasmonic acid (JA) signalling responsible for digestive enzyme secretion. Mechanical stimulation of trigger hair under diethyl ether (DE) anaesthesia still induces increased [Ca^2+^]_cyt_ in podium cells, however, the Ca^2+^ and electric signals can not escape from trigger hair. Thus, AP is not generated in trap lobe and JA signalling is not activated. The trap is unresponsive to insect prey, it cannot snap shut and activate the digestive process (according to Pavlovič et al. 2020; Scherzer et al. 2022a, b)

Plants are very sensitive to touch and their responses to mechanical stimuli are very diverse despite similar molecular mechanisms (Pavlovič, 2022). Only certain plants have rapid and noticeable touch-stimulus responses observable by the naked eyes (e.g. seismonastic sensitive plant *Mimosa*, carnivorous plants *Drosera* and *Dionaea*). Although not all plants have the ability to move, the rapid response to touch detected as changes in gene expression is obviously present in all of them (Matsumura et al. 2022). Also plants without specialized sensory cells react to mechanical stimuli slowly over time by altering their morphology as well as their growth rate and this response is called thigmomorphogenesis (Chehab et al. 2009). Because long-term exposure of plants to general anaesthetics may have damaging effect (Lin et al. 2011; Tseng and Lin 2016), mainly rapid responses have been studied so far. Already Charles Darwin (1875) observed anaesthetic effect of chloroform and ether on tentacle bending reaction in the carnivorous sundew plant *Drosera rotundifolia* and trap closing reaction in the Venus flytrap (*Dionaea muscipula*). Claude Bernard (1878) observed complete immobilization of *Mimosa pudica* leaves in response to exposure of ether vapour. All these plants lost ability to move with their leaves in response to touch after exposure to general volatile anaesthetics (GVAs), but this ability was recovered after removing of anaesthesia within minutes. Interestingly, *Mimosa*, but not *Dionaea*, is sensitive to local anaesthetic lidocaine applied through the roots (Milne and Beamish 1999; De Luccia 2012). This finding is interesting, because plants do not have specific animal-type sodium channels (Hedrich 2012), indicating also some general target of local anaesthetic. To reveal the possible mechanism responsible for the complete immobilization of leaves, Yokawa et al. (2018) and Pavlovič et al. (2020) found that diethyl ether anaesthesia inhibits the generation and propagation of action potentials in the trap of Venus flytrap, what strongly resembles the effect of anaesthesia on animal neurons. Recent success in the transformation of the Venus flytrap with calcium reporter system GCaMP6f (Suda et al. 2020) has allowed monitoring of spatio-temporal dynamics of intracellular calcium ion ([Ca^2+^]_cyt_), one of the components of action potential in the Venus flytrap (Scherzer et al. 2022a). Mechanical stimulation of a trigger hair causes an increase in cytosolic [Ca^2+^]_cyt_ starting in the podium cells of trigger hair and spreading concentrically to the trap blade. Using these plants, Scherzer et al. (2022b) showed that Ca^2+^ signal from the leaf blade was inhibited by diethyl ether anaesthesia but the signal from podium cells was not. This indicates that the plant can sense mechanical stimulation under diethyl ether anaesthesia probably through the stretch-activated channels (e.g. HYPEROSMOLARITY-GATED CALCIUM-PERMEABLE CHANNEL, OSCA) but is not able to send electrical and Ca^2+^ signals to the effector – trap lobes, thus trap closing response is blocked (Fig. 1). Based on the sequenced RNA expressed in the trigger hair and ability of glutamate to induce increase of [Ca^2+^]_cyt_, which is supressed by diethyl ether, Scherzer et al. (2022b) have concluded that GLUTAMATE RECEPTOR-LIKE 3.6 (DmGLR3.6) is targeted by diethyl ether. Because a tight link between electrical and jasmonic acid (JA) signalling pathway exists (Farmer et al. 2020), the Venus flytrap is not able to activate the JA signalling pathway necessary for the activation of the digestive process under anaesthesia (Pavlovič et al. 2020). In this way, the mechanosensing ability of the Venus flytrap is not lost under diethyl ether anaesthesia, because the plants is still able to sense mechanical stimulation in podium cells of trigger hairs. Only the signal transduction is affected and the Venus flytrap cannot capture and digest insect prey under diethyl ether anaesthesia.

The ability to sense touch under diethyl ether anaesthesia was recently found also in *Arabidopsis thaliana*. Under anaesthesia, the leaves can still increase local [Ca^2+^]_cyt_ in response to touch and activate the expression of touch-responsive JA-independent genes (*TCH3,* *TCH4, CML23*) and JA-dependent genes (*JAZ7*, *JAZ10*, Hřivňacký et al. 2024). This indicates that stretch-activated channels (e.g. OSCA) are probably not inhibited by GVA. If we consider GLR receptors as one of the possible targets of GVA (Jakšová et al. 2021; Scherzer et al. 2022a, b), it is in line with evidence that GLR receptors are not or only redundantly essential for touch response (Darwish et al. 2022; Pantazopoulou et al. 2023). However, the touch-responsive JA-independent genes were also upregulated by the application of diethyl ether without any previous mechanical stimuli (Hřivňacký et al. 2024). The touch-responsive genes are often upregulated by stimuli which share no mechanical properties like darkness, cold and heat stress (Chehab et al. 2009). Because the application of diethyl ether induced increase [Ca^2+^]_cyt_ and partially mimicked the heat stress (Pavlovič et al. 2022; Hřivňacký et al. 2024), it is not surprising that it also upregulated touch-responsive JA-independent genes *TCH2*, *TCH4, CML23* without any mechanical stimuli. In this case, diethyl ether partially mimicked Ca^2+^ dependent and JA-independent touch response.

Algae like *Chara* are also sensitive to touch. They respond to mechanical stimulation by the generation of action potentials and immediately halting cytoplasmic streaming. The increased [Ca^2+^]_cyt_ inhibits the interaction of actin and myosin, thus inhibiting the proteins that provide the motive force for streaming (Tominaga and Ito 2015). Recently Rodgers and Staves (2024) found that *Chara* cells anaesthetized with GVA chloroform did not stop cytoplasmic streaming upon mechanostimulation. But because the authors did not measure electrical signals or [Ca^2+^]_cyt_ in response to GVA application, it is difficult to judge if sensing (e.g. [Ca^2+^]_cyt_) or signal transduction element is affected.

### Defence response

**Fig. 2:**
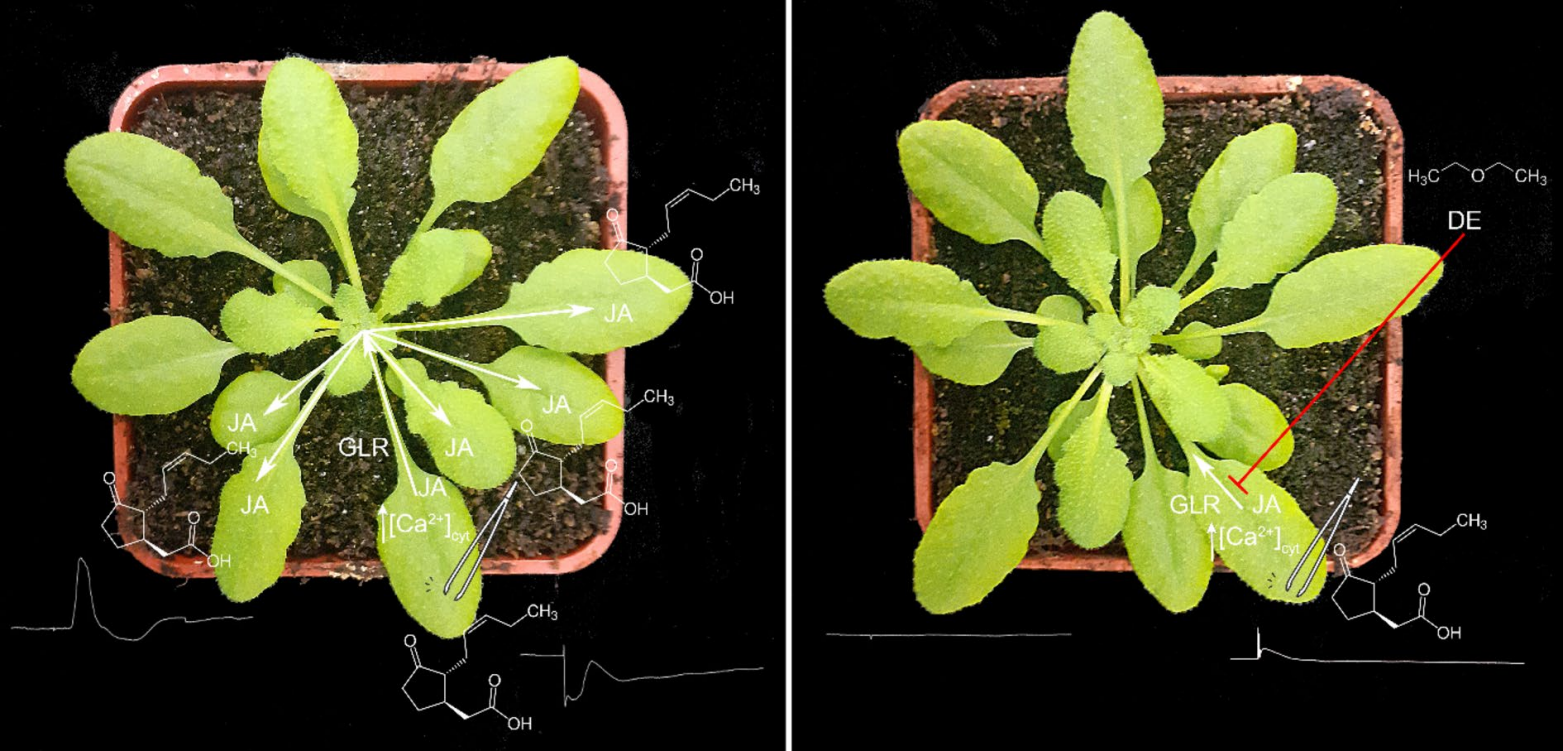
The systemic response in *Arabidopsis thaliana* is suppressed by diethyl ether (DE) anaesthesia. Leaf wounding of local leaf triggers increased intracellular calcium level [Ca^2+^]_cyt_ and electrical signals which propagate to neighbouring systemic leaves. The propagation is dependent on GLR channels. All leaves which receive these signals accumulate jasmonic acid (JA) which triggers defence response (plant on the left). But when the plants were exposed to DE, elevated [Ca^2+^]_cyt_ signal did not propagate to neighbouring systemic leaves and was locked in the local leaf. Accumulation of JA and defence response was activated only in the local leaf (plant on the right, according to Jakšová et al. 2021)

Touch signal is often considered only as the first alert for activation of defence response and may prime the plants for potential herbivore attack later (Chehab et al. 2009; Moerkercke et al. 2019; Matsumura et al. 2022). However, the full activation of defence response starts after the physical disruption of leaf tissue integrity (Maffei et al. 2007). Wounding freed lipids from the cell membranes of damaged leaves by phospholipase A-Type 1 (PLA1) and alpha-linoleic acid (α-LA) is synthetized which is further metabolized to the phytohormone JA, (Kimberlin et al. 2022). Local wounding leads to a burst in newly synthesized JA not only in local leaves but also in leaves distal to wounds activating a systemic response. The signals responsible for systemic JA accumulation in distal leaves of experimental model plant *A. thaliana* are electrical signals (Mousavi et al. 2013), Ca^2+^ wave (Toyota et al. 2018), reactive oxygen species (ROS, Miller et al. 2009; Suzuki et al. 2013) and/or JAs (Li et al. 2020), and the signals co-propagate together (Gilroy et al. 2014). In the nucleus, the bioactive isoleucine conjugate of JA (JA-Ile) triggers an interaction between the CORONATINE INSENSITIVE1 (COI1) receptor and members of the JASMONATE ZIM-DOMAIN (JAZ) family of repressors. COI1-mediated degradation of JAZ repressors activates the reprogramming of gene expression leading to plant defence response (Chini et al. 2007; Thines et al. 2007; Fonseca et al. 2009; Sheard et al. 2010). Diethyl ether anaesthesia was not able completely supressed wound response in the local wounded leaf of Venus flytrap and *A. thaliana* (Pavlovič et al. 2020; Jakšová et al. 2021). Wounded leaf of *A. thaliana* still showed increased [Ca^2+^]_cyt_, accumulated JA and JA-Ile, and activated expression of JA-responsive genes but only to app. 25% of non-anaesthetized wounded leaf. However, anaesthesia was able completely blocked electrical and Ca^2+^ signal propagation to systemic leaves and the plants were not able to induce JA-dependent systemic defence response (Fig. 2). Systemic electrical and Ca^2+^ signal propagation is dependent on GLR3.3 and GLR3.6 and exogenous application of glutamate can mimic wounding (Mousavi et al. 2013; Toyota et al. 2018). Glutamate is released at the site of wounding and propagates through vasculature probably by bulk flow activating Ca^2+^ permeable GLR channels (Bellandi et al. 2022) or is released locally as a result of increased turgor pressure (Grenzi et al. 2023). Because diethyl ether anaesthesia was able to block also systemic response evoked by exogenous glutamate application, it is tempting to assume that plant GLRs are among the targets of diethyl ether anaesthesia in plants (Jakšová et al. 2021; Scherzer et al. 2022a, b). The fact that plants use homologous proteins to animal ionotropic glutamate receptors (iGluRs) (Lam et al. 1998), which serve for nerve impulse transmission and both are sensitive to general anaesthetics, is amazing. However, the recent study has shown that electrical signal propagation in plants is very complex. Gao et al. (2023) showed that after wounding, myrosinase enzyme and its thioglucoside substrates are released from idioblasts and migrate through the vasculature. The enzyme hydrolyses thioglucoside to aglucone, elicitor of membrane depolarization and JA response in leaves distal to wound (Gao et al. 2023). Existence of such chemical elicitor was predicted a long time ago by Ubaldo Ricca (known as Ricca’s factor, Ricca 1916). How this system interacts with glutamate-dependent electrical signal propagation is not known but both are attenuated in *glr3.3 glr3.6* double mutant. We cannot exclude the possibility that GVA diethyl ether affects GLR channels only indirectly by interacting with the complicated interplay of different cellular factors.

In contrast to diethyl ether, ketamine, a well-known intravenous general anaesthetic, antagonist of NMDA receptors, an ionotropic glutamate receptor, did not block systemic reaction in *A. thaliana*, only modified the characteristics of electrical signals by decreasing the amplitude and duration (Pavlovič et al. 2024b). The same anaesthetic had no effect on trap closure in Venus flytrap (de Luccia 2012). Recently, cryo-electron microscope structures of human NMDA receptors in complex with ketamine was resolved (Zhang et al. 2021). The structure revealed two important amino acid as key residues that form hydrophobic and hydrogen-bound interactions with ketamine. Both amino acids are not conserved in plant GLRs, what may explain the low potency of ketamine in plants (Pavlovič et al. 2024b). In addition to its action on NMDA receptors, alternative targets for ketamine probably also exist and must be considered (Zorumski et al. 2016; Yin et al. 2019). Recently, Sylvain-Bonfanti et al. (2024) found that local anaesthetic lidocaine decreased amplitude of electrical signals in rice and tobacco seedlings in response to leaf burning. This finding is interesting because lidocaine as a local anaesthetic blocks voltage-gated sodium channels (VGSCs) leading to a reversible block of action potential propagation and plants do not have any voltage-gated Na^+^ channels (Hedrich 2012). These studies on plants have confirmed that even anaesthetics with well-known defined targets have an off-target effect. In the case of lidocaine, such targets involve potassium and calcium channels and even NMDA receptors but usually in above clinically relevant plasma concentrations (Hermanns et al. 2019).

### Light response

**Fig. 3:**
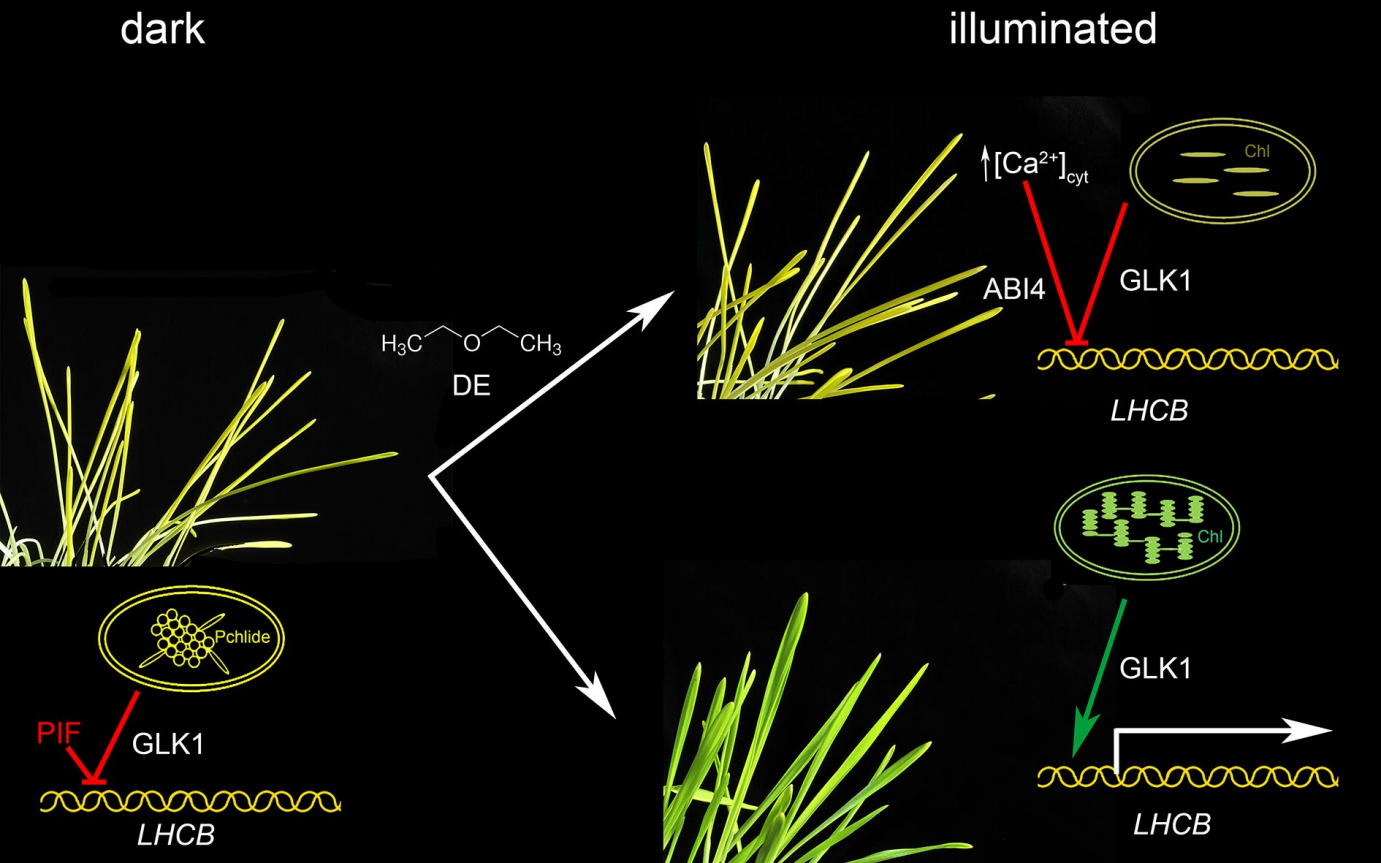
Inhibited de-etiolation in barley (*Hordeum vulgare*) by diethyl ether (DE) anaesthesia. Dark-grown etiolated barley seedlings contain etioplasts which after exposure to light transform to photosynthetic active chloroplasts. During this process, negative regulators of photomorphogenesis (e.g. PIFs) are proteolytically degraded in response to phytochrome migration into the nucleus. Concomitantly, positive regulators accumulate in response to phytochrome action (e.g. HY5) as well as positive retrograde signals from plastids (e.g. through GLK1). These factors trigger the expression of nuclear-encoded *LHCB* genes. Exposure of plants to diethyl ether locked the plants in intermediate skoto-photomorphogenetic state. They were not able to form functional photosystems and accumulated only tiny amount of chlorophylls. Such dysfunctional plastids emitted negative retrograde signals to nucleus (e.g. through GLK1) and inhibited the expression of *LHCB* genes. Moreover, elevation of [Ca^2+^]_cyt_ in response to diethyl ether application might also inhibit *LHCB* expression through phosphorylation of ABI4. *Pchlide* protochlorophyllide, *Chl* chlorophyll (according to Pavlovič et al. 2024a, b)

Light is essential for plants not only for photosynthesis but it is also very important environmental stimulus which triggers light-induced development called photomorphogenesis. Plants sense light using a variety of photoreceptors including phytochromes, cryptochromes and phototropins (Möglich et al. 2010). In the dark, a type of growth called skotomorphogenesis is coupled with the etiolation phenomenon in angiosperms. Etiolation involves prolonged growth in the absence of light that results in the development of etioplasts instead of chloroplasts. Etioplasts do not contain chlorophylls, thylakoid membranes and photosystems as chloroplasts do but rather have a paracrystalline structure known as the prolamellar body (PLB). When dark-grown plants are exposed to light, a series of physiological and biochemical changes occur. This transition, called de-etiolation or simply as greening, is coupled to a light-driven etioplast-to-chloroplast transition and chlorophyll biosynthesis (Kanervo et al. 2008; Armarego-Marriott et al. 2020). Exposure of etiolated barley plants (*Hordeum vulgare*) to diethyl ether inhibited the process of de-etiolation and the plants were locked in intermediate skoto-morphogenetic state (Yokawa et al. 2018; Pavlovič et al. 2024a). Plants were able to synthetize limited amount of chlorophylls, because light-driven photoenzyme protochlorophyllide oxidoreductase (POR) was not inhibited and could still photoreduce precursor molecule of protochlorophyllide to chlorophyllide. However, the expression of light-induced nuclear genes encoding pigment-protein complexes and enzymes of chlorophyll biosynthesis were strongly attenuated as well as assembly of functional photosynthetic apparatus. Indirect evidence indicates, that the plant was still able to sense light through phytochrome photoreceptor under diethyl ether anaesthesia, but the response to light was blocked (Fig. 3). One of the candidates, which blocks expression of nuclear-encoded light-responsive genes, is a negative (or inhibition of positive) signal from plastid to nucleus (i.e. retrograde signal), which do not allow de-repression of nuclear-encoded genes in response to light (Pavlovič et al. 2024a).

### Germination and circumnutation movement

Anaesthesia with diethyl ether, ethyl vinyl ether, xenon and lidocaine inhibited seed germination (Bernard 1878; Grémiaux et al. 2014; Yokawa et al. 2018). Autonomous circumnutation of tendrils in pea plants was stopped immediately after exposure to diethyl ether and were immobilized in a curled shape (Yokawa et al. 2018). This is probably not such surprising as glutamatergic elements are probably involved in circumnutation mechanism (Stolarz et al. 2010).

### Diethyl ether anaesthesia as stress factor

Besides anaesthetic effect, there is also another view on diethyl ether in plant studies; as a stress factor from the group of volatile organic compounds (VOC, Lin et al. 2011; Tseng and Lin 2016). Long-term 24-h incubation of tomato plants in a diethyl ether atmosphere resulted in dead cell and the plants died quickly (Lin et al. 2011). Arabidopsis plants were somehow more resistant to the toxic effect of ether (Tseng and Lin 2016). When the diethyl ether vapour was allowed to evaporate directly under the trap of Venus flytrap it induced rapid chemonastic trap closure (Volkov et al. 2014). Local anaesthetic lidocaine at 1% concentration also induced programmed cell death (Sylvain-Bonfanti et al. 2024). So, as in the human anaesthesiology, concentration, type of application and time duration matter. Application of diethyl ether induced strong reprogramming of gene expression in *A. thaliana*. The RNA-seq analysis showed that more than half out of 21,500 genes were either up- or down-regulated. The gene ontology (GO) analysis revealed the downregulation of genes encoding enzymes of tetrapyrrole biosynthesis and photosynthesis and upregulation of genes involved in heat stress, vesicle-mediated transport, calcium signalling or response to oxidative stress etc. (Pavlovič et al. 2022; 2024a). This is in line with evidence that GVA anaesthesia inhibited photosynthesis, chlorophyll accumulation, affects endocytic vesicle recycling, and induces reactive oxygen species (ROS, Nakao et al. 1998; Lin et al. 2011; Tseng and Lin 2016; Yokawa et al. 2018; 2019). Using transgenic *A. thaliana* expressing apoaequorine, we also showed that the application of diethyl ether increased [Ca^2+^]_cyt_ (Pavlovič et al. 2022; Hřivňacký et al. 2024). This [Ca^2+^]_cyt_ has different spatio-temporal pattern in comparison to wound-induced [Ca^2+^]_cyt_, which is effectively blocked by diethyl ether anaesthesia (Jakšová et al. 2021), indicating a different effect of diethyl ether on the two classes of Ca^2+^ ion channels. This elevated [Ca^2+^]_cyt_ is probably responsible for huge reprogramming of gene expression. Previously, it was shown that preceding Ca^2+^ transient through Ca^2+^ permeable channel, which is activated by heat but also membrane fluidizers, was responsible for increased expression of HSPs, which were also strongly induced by diethyl ether anaesthesia (Saidi et al. 2009; Pavlovič et al. 2022). This indicates that diethyl ether anaesthesia partially mimics heat stress through the effect on membrane fluidization and Ca^2+^ signalling (Pavlovič et al. 2022), but increased temperature cannot mimic anaesthesia, what is one of the argument against lipid hypothesis of general anaesthesia (Franks 2006). Increased membrane fluidity caused by GVA compensated increasing membrane rigidity with decreasing temperature and reduced chilling injury in plants (Saltveit et al. 1993). Upregulation of heat shock response by general anaesthetic protected plants against subsequent heat stress through the effect of cross-protection or priming (Pavlovič et al. 2022). Application of lidocaine also alleviated hyperosmotic and hypoosmotic stress probably through alternationation of Ca^2+^ signalling (Sylvain-Bonfanti et al. 2024). Thus, GVA may be helpful for plants to better withstand abiotic stresses. Such anaesthetic preconditioning is known also in animal anaesthesia (Zaug et al. 2003; Sergeev et al. 2004).

## Concluding remarks

It seems that plants can sense diverse stimuli under general anaesthesia (mechanical stimuli, wounding, light) but in the case when the response involves transduction of information or signalling, they cannot respond to them. Anaesthesia in plants disrupts one-way information exchange between different organs (wound response in *Arabidopsis,* from leaf to leaf), tissues (touch response in *Dionaea*, from trigger hair to trap lobe) and affects also information exchange between organelles (light response in *Hordeum*, from chloroplast to nucleus). If you apply touch, wound or heat stimuli to your skin, you will not be able to realize them under anaesthesia because of inhibited nerve impulse transmission from your sensory neurons (nociceptors) to the spinal cord and neurons in the brain, where sensory signals are processed and interpreted; you lose the consciousness. But your skin cells probably will sense the stimuli even under anaesthesia, like the plant cells do, but they cannot send this information to other cells. This is what general anaesthetics have common in animals, humans and plants. This is probably due to the general non-specific effects of anaesthetics on proteins and/or lipids. Regardless of the fact that plants use the same class of glutamate receptors to transmit electrical signals as animals (Mousavi et al. 2013; Toyota et al. 2018), many proteins have hydrophobic cavities which can be occupied by anaesthetics and thus may have altered function. Presynaptic neurotransmitter release in animal neurons rely on the same set of proteins (i.e. SNARE complex) which are responsible for vesicle transport in plants, and both are sensitive to anaesthesia (van Snideren et al. 2014; Yokawa et al. 2018). Dynamic reorganization of actin filaments is important for seismonastic movement in plants (Kanzawa et al. 2006). All the possible targets of general anaesthesia listed above (proteins, lipids, vesicles, cytoskeleton and mitochondria) are not mutually exclusive and may contribute to a similar anaesthesia effect in animals and plants. There are still large gaps in our understanding of how anaesthesia works in plants and more detailed studies are necessary. Only response to touch, wounding and light have been investigated in details but how anaesthesia affect other plant reactions like e.g. gravitropism, phototropism or flower induction? Induction of [Ca^2+^]_cyt_ signature by GVA and activation of stress response prime plants to better withstand subsequent stress. How can we transfer this information to horticultural practice to have plants better acclimated to chilling or heat stress? Plants can also synthetize anaesthetic compounds. What is their eco-physiological significance? Recent successful transformation of GLRs channel into HEK292T cells (Shao et al. 2020) may enable evaluate the direct effect of anaesthetics by patch-clamp recordings of the plant channel activities. Many anaesthetic substances exist and are waiting to be tested for their effect on plants.
